# The lncRNA-mediated ceRNA network of *Altica viridicyanea* is involved in the regulation of the Toll/Imd signaling pathway under antibiotic treatment

**DOI:** 10.3389/fphys.2023.1244190

**Published:** 2023-08-17

**Authors:** Yipeng Ren, Juhong Chen, Yuan Wang, Siying Fu, Wenjun Bu, Huaijun Xue

**Affiliations:** Institute of Entomology, College of Life Sciences, Nankai University, Tianjin, China

**Keywords:** *Altica viridicyanea*, long noncoding RNAs, Toll/Imd signaling pathway, gut bacterial microbiota, competing endogenous RNA

## Abstract

Long noncoding RNAs (lncRNAs) play significant roles in the regulation of mRNA expression or in shaping the competing endogenous RNA (ceRNA) network by targeting miRNA. The insect gut is one of the most important tissues due to direct contact with external pathogens and functions in the immune defense against pathogen infection through the innate immune system and symbionts, but there are limited observations on the role of the lncRNA-involved ceRNA network of the Toll/Imd pathway and correlation analysis between this network and bacterial microbiota in the *Altica viridicyanea* gut. In this research, we constructed and sequenced six RNA sequencing libraries using normal and antibiotic-reared samples, generating a total of 17,193 lncRNAs and 26,361 mRNAs from massive clean data by quality control and bioinformatic analysis. Furthermore, a set of 8,539 differentially expressed lncRNAs (DELs) and 13,263 differentially expressed mRNAs (DEMs), of which related to various immune signaling pathways, such as the Toll/Imd, JAK/STAT, NF-κB, and PI3K-Akt signaling pathways, were obtained between the two experimental groups in *A. viridicyanea*. In addition, numerous GO and KEGG enrichment analyses were used to annotate the DELs and their target genes. Moreover, six *Toll* family members and nineteen signal genes from the Toll/Imd signaling pathway were identified and characterized using online tools, and phylogenetic analyses of the above genes proved their classification. Next, a lncRNA-miRNA-mRNA network of the Toll/Imd pathway was built, and it contained different numbers of DEMs in this pathway and related DELs based on prediction and annotation. In addition, qRT-PCR validation and sequencing data were conducted to show the expression patterns of the above DELs and DEMs related to the Toll/Imd signaling pathway. Finally, the correlated investigations between DELs or DEMs of the Toll/Imd signaling pathway and most changes in the gut bacterial microbiota revealed significantly positive or negative relationships between them. The present findings provide essential evidence for innate immune ceRNAs in the beetle gut and uncover new potential relationships between innate immune pathways and the gut bacterial microbiota in insects.

## 1 Introduction

Animals, both vertebrates and invertebrates, have evolved a variety of strategies to recognize and eradicate invading microbes, including innate and adaptive immunity. Due to the lack of an adaptive immune system, insects mainly depend on the innate immune system, a nonspecific immunity that could provide the first line of defense against infections through pattern recognition receptors (PRRs), such as Toll receptors, peptidoglycan-recognition proteins (PGRPs), gram-negative bacteria-binding proteins (GNBPs), and scavenger receptors (SRs) (Myllymaki et al., 2014; Zhang et al., 2022a). Overall, insects can activate the humoral response against pathogens by secreting antimicrobial peptides (AMPs) and initiate the cellular response to fight infections by phagocytosing bacteria and encapsulating parasites (Myllymaki et al., 2014). To date, the production of AMPs through Toll and Imd (immune deficiency) signaling pathways via NF-κB transcription factors has been well investigated in model insects against either gram-positive bacterial/fungal or gram-negative bacterial infections (Silverman et al., 2009; Valanne et al., 2022). In detail, bacterial and fungal structures, with specific pathogen-associated molecular patterns (PAMPs), were recognized and then active ligand Spaetzle to form dimers and bind to Tolls, which is the first step that allows signal transduction into the cell. Intracellular signaling factors, including MyD88, Tube, and Pelle, are combined, and finally, Pelle results in the phosphorylation and degradation of Cactus, which releases two NF-κB transcription factors, Dorsal and dorsal-related immunity factor (Dif), to promote AMP expression (Lemaitre and Hoffmann, 2007; Valanne et al., 2022). Likewise, the transmembrane receptor PGRP-LC leads to recruitment of a signaling complex consisting of Imd, the adaptor protein Fadd, and Dredd, and then the TAB2/TAK1 complex can phosphorylate and activate the IKK complex and finally regulate the activation of Relish, whose N-terminal part (Rel-68) ultimately translocates into the nucleus to promote the transcription of AMP genes in *Drosophila*, such as *Diptericin* and *Cecropin* (Myllymaki et al., 2014).

With the development of high-throughput sequencing technology, RNA sequencing (RNA-seq)-based transcriptome analysis is a useful tool and has been conducted extensively to understand the developmental function or immune responses in insects (Wu et al., 2016a; Chang et al., 2018; Yang et al., 2022). Noncoding RNAs (ncRNAs), categorized as microRNAs (miRNAs), small interfering RNAs (siRNAs), PIWI-interacting RNAs (piRNAs), circular RNAs (circRNAs) and long noncoding RNAs (lncRNAs), are supposed to be “transcriptional noise” owing to their transcription from DNA without, protein-coding functions but are actually functional RNA molecules that play essential roles in regulating mRNA expression (Alexander et al., 2010; Cech and Steitz, 2014). Among these ncRNAs, lncRNAs, longer than 200 nucleotides (nt), are usually divided into exonic, intronic, intergenic, and overlapping lncRNAs based on their location relative to protein-coding transcripts (Quinn and Chang, 2016). Over the past decade, increasing evidence has suggested that they are widely expressed in various tissues and exert diverse biological functions by influencing the stability and translation of mRNAs and then activating or deactivating downstream signaling pathways (Statello et al., 2021). For instance, the lncRNAs CR11538, CR46018 and CR33942 have been shown to prevent or initiate the transcription of immune effectors in the Toll/Imd signaling in *Drosophila* (Zhou et al., 2021a; Zhou et al., 2021b; Zhou et al., 2022a). Besides, a hot topic is that lncRNAs or circRNAs are regarded as competitive endogenous RNAs (ceRNAs) or miRNA sponges, which could participate in competitively inhibiting miRNAs to bind target genes (Pasquinelli, 2012). To the best of our knowledge, the gut microbiota serves as a complex and dynamic biological system composed of commensal, symbiotic and pathogenic microbial communities, which contribute to host nutrient absorption and energy metabolism and improve host fitness (Evariste et al., 2019). Therefore, it has been demonstrated that gut flora health is largely beneficial to overall host wellbeing. Conversely, an imbalance in gut microbiota composition, termed dysbiosis, may cause host fitness loss and increase susceptibility to pathogens (Zhang et al., 2015). However, there are a limited number of studies on the regulatory roles of ceRNAs under the disturbance of gut microbiome homeostasis using antibiotic treatment in beetles.

*Altica viridicyanea*, the leaf beetle genus *Altica*, is regarded as a good model to investigate plant secondary chemistry metabolism by gut microbes based on diversification in its host plant, *Geranium nepalense* Sweet (Geraniaceae) (Wei et al., 2022). In the present study, we performed RNA library construction and sequencing from normal and antibiotic-reared samples, which produced massive lncRNA and mRNA sequences after quality control and filtration using the MGI2000 platform. Moreover, the characterization, differentially expressed analysis and annotation information of the above data were executed based on bioinformatic analysis between the two experimental groups. Subsequently, we transcriptome-wide identified and characterized the cDNA and protein features of Toll/Imd signaling pathway genes. In addition, the expression models of differentially expressed mRNAs (DEMs) from the above genes and their related differentially expressed lncRNAs (DELs) were selected and calculated according to the transcriptome data and qRT‒PCR validation. Finally, a lncRNA-associated ceRNA regulatory network of the Toll/Imd signaling pathway in the *A. viridicyanea* gut was successfully established, and we applied correlation analysis between DEMs or DELs from the Toll/Imd signaling pathway and most changes in the gut bacterial microbiota from previous 16S rRNA amplicon sequencing to investigate the disturbance in the gut bacterial microbiota in *A. viridicyanea* under antibiotic treatment. The combined multiomics data were used to explore the innate immune ceRNA network and interplay of the gut bacterial microbiota and Toll/Imd signaling pathway at the coding and noncoding RNA levels, which will pave new avenues for further observations of host-microbiota interactions and adaptation in insects.

## 2 Materials and methods

### 2.1 Sample collection and treatment

All animal experimental procedures were approved by the Animal Care and Use Committee of Nankai University. In this study, samples of *A. viridicyanea* individuals were collected from Jizhou district (40.22′N, 117.50′E) in Tianjin, China on 19th May 2022 and were reared in an environmentally controlled growth chamber maintained at 25°C temperature with 60% relative humidity and a 16 h: 8 h (L:D) photoperiod, feeding on *Geranium nepalense* Sweet (Geraniaceae). Moreover, newly born eggs and hatched larvae of *A. viridicyanea* were obtained and kept in 90 mm diameter Petri dishes padded with moist filter papers, and then, hatched second larval instars were fed with normal host leaves or with those by antibiotic treatment until sexual maturation (10 days after emerging adults), as described in our previous report (Wei et al., 2020). In brief, 5 mL of antibiotic solution (50 μg/mL tetracycline, 200 μg/mL rifampicin, 100 μg/mL streptomycin = 1:2:4) was used to completely submerged leaves for 5 min. Then, a total of 50 normal (C group) and 50 antibiotic-reared individuals (A group), starved after 2 days, were used to gently dissect the whole gut tissues by clamping the head of living beetles with sterile forceps and removing the head. Finally, ten guts from the C or A group were surface-sterilized in 70% ethanol for 1 min, randomly pooled to build the transcriptome library as one replicate, immediately frozen in liquid nitrogen and stored at −80°C until subsequent total RNA extraction. In addition, the remaining guts after library construction were used for qRT-PCR analysis.

### 2.2 RNA extraction and library construction

In this study, transcriptome sequencing was performed at Beijing Genomics Institution (BGI; Wuhan, China) Technology Co., Ltd. with three biological replicates of each treatment. Total RNA of all experimental guts was isolated using TRIzol reagent (Invitrogen, United States) according to the manufacturer’s protocols. In addition, RNA purity was assessed using the OD260/OD280 absorbance ratio between 1.8 and 2.0. RNA quality was visualized by 1% agarose gel electrophoresis staining, and RNA quantity was measured with an Agilent 2,100 Bioanalyzer (Agilent Technologies, United States). Finally, 5 μg of total RNA was removed from ribosomal RNAs (rRNAs) and used to prepare the transcriptome library construction.

Briefly, first-strand cDNA was produced with random hexamer primers and M-MuLV Reverse Transcriptase, and then the second strand was synthesized by adding RNaseH, dNTPs, DNA polymerase I, and buffer. Subsequently, the product was purified with AMPure XP beads, the cohesive ends of DNA were repaired using T4 DNA polymerase and Klenow DNA polymerase, and the product was further size-selected and ligated to an adaptor. Furthermore, we purified and amplified each product by AMPure XP beads and PCR (polymerase chain reaction), respectively. At the last step for preparation of the final sequencing library, magnetic beads were used to capture the amplified cDNA fragments, and the quality of all libraries was assessed using a Agilent BioAnalyzer 2,100 system (Agilent Technologies, United States). Later on, the diluted 1 ng/μL cDNA with an average insert size of 250–300 bp (base pair) was loaded for 150 bp paired-end sequencing using the MGI2000 platform.

### 2.3 Quality control, assembly and mapping of transcriptome data

The raw reads with adaptor contamination, low-quality, and undetermined data were removed using SOAPnuke software (version 1.5.2) to produce clean reads, and the Q20, Q30, and GC contents, representing effective sequencing values, were calculated. Furthermore, the quality of clean reads was estimated by FastQC (version 0.10.1), and then we mapped clean data to the genome of *A. viridicyanea* (data unpublished) with Hisat 2 (version 2.0.4) (Kim et al., 2019). The mapped reads of each group were assembled by StringTie (version 1.0.4), and finally, the transcriptome results were outputted (Pertea et al., 2015).

### 2.4 Identification of mRNAs and lncRNAs

First, we applied Cufflinks software (version 2.2.1) to cover the transcripts with a clear chain orientation, and subsequently, the protein coding potentials were found by screening transcripts with no fewer than 2 exons and more than 200 bp in length. Moreover, CNCI software (CNCI_threshold >0 is mRNA, CNCI_threshold < 0 is lncRNA) was used to classify the coding transcripts and noncoding transcripts based on the spectrum of adjacent trinucleotides (Sun et al., 2013). Next, we used CPC (version 0.9-r2) (CPC_threshold >0 is mRNA, CPC_threshold < 0 is lncRNA) and txCdsPredict (txCdsPredict_threshold >500 is mRNA, txCdsPredict_threshold < 500 is lncRNA) software to assess the transcripts with coding potential according to the biological sequence characteristics (Kong et al., 2007). In addition, Pfam_Scan software was used to select the known protein family domains (Finn et al., 2010). In our study, the selected principle is that only transcripts identified by three of the above four methods were considered coding mRNAs or lncRNAs (Zhang et al., 2022b).

### 2.5 Transcription analysis of mRNAs and lncRNAs

The expression levels of total mRNAs and lncRNAs were calculated and normalized to transcripts per million (TPM). In addition, RPKM (reads per kilobase of exon model per million mapped reads) was conducted to estimate their expression abundance in different samples, and the infrequently expressed transcripts with FPKM < 1 in all samples were filtered out. Furthermore, the correlation and principal component analysis (PCA) analyses based on RPKM using StringTie (version 1.0.4) software, and then the significantly differentially expressed mRNA and lncRNA were considered with absolute Log_2_ | Fold Change | >1 and *p*-value < 0.001 by edgeR (version 3.14.0) software (Love et al., 2014).

### 2.6 Functional enrichment of transcriptome data and target prediction

First, to obtain annotation information on the functions of obtained mRNAs, all of them were searched against the Nr (NCBI nonredundant protein sequences), Nt (NCBI nucleotide), SwissProt (a manually annotated and reviewed protein sequence database), KOG (eukaryotic Ortholog Groups), and KEGG (Kyoto Encyclopedia of Genes and Genomes) databases using BLASTX (basic local alignment search tool) and BLASTN with an E-value < 10^–5^. In addition, Blast2GO was used for GO (Gene Ontology) annotation with an E-value < 10^–5^ from the Nr annotation results (Fu et al., 2021).

Moreover, the DELs and their target genes were functionally annotated by GO and KEGG databases. The GOseq R package was used to execute the GO enrichment, and KOBAS was used to explore the KEGG enrichment (Yu et al., 2012). The statistical significance between the A and C groups was considered the threshold to select significantly enriched GO terms and KEGG pathways (*p*-value < 0.01).

To explore the regulatory function of lncRNAs, *cis*/trans-regulatory algorithms were applied to predict the target genes. First, the regions 0–10 kb upstream or 0–20 kb downstream of *cis*-acting lncRNAs were screened to identify colocated target genes, and then the lncRNAs were considered trans-acting on the target genes once the binding energy was < −30 and exceeded the above region. Meanwhile, Pearson ≥0.6 and Spearman ≥0.6 correlation coefficients between the lncRNAs and target genes were used to predict the coexpressed target genes. In addition, the overlaps between the lncRNAs and target genes were classified into four categories: overlap, anti-overlap, incomplete, and anti-incomplete (Zhang et al., 2022b).

### 2.7 Identification and characterization of Toll/Imd signaling pathway genes

To identify candidate cDNA sequences of the Toll/Imd signaling pathway from sequencing data in *A. viridicyanea*, the published above genes from insects were used to search against the available information using TBLASTN and BLASTP databases with E-values < 0.001 as the threshold to find the corresponding gene members (Tong et al., 2015). Furthermore, the ORFs (open reading frames) of the putative cDNA sequences were translated using the ORF finder server, and the conserved domains of all the protein sequences were predicted by the SMART (simple modular architecture research tool) and TMHMM (version 2.0) servers. The signal peptide (SP) was examined using the program SignalP (version 5.0). In addition, for the protein sequence of each gene, basic information, such as molecular weight, length of protein, and isoelectronic point (PI), was identified with the ExPASy ProtParam server (Ren et al., 2021). Later on, the subcellular localization of the six *Toll* genes was predicted with WoLF PSORT (Zou et al., 2023). Lastly, MAFFT and MEGA (version X; Molecular Evolutionary Genetics Analysis) programs were applied to execute multiple sequence alignment of the amino acid sequences of the Toll/Imd signaling pathway genes from *D. melanogaster*, *Anopheles gambiae*, *Bombyx mori*, *Tribolium castaneum*, *Diabrotica virgifera virgifera* and *A. viridicyanea*, and to construct phylogenetic trees with the neighbor-joining (NJ) method (1,000 replicate bootstraps), respectively (Supplementary Table S2). Meanwhile, the results of phylogenetic analysis were optimized and shown using FigTree software (Tong et al., 2015). In this study, the obtained complete cDNA sequences of Toll/Imd signaling pathway genes were submitted to the GenBank database.

### 2.8 The ceRNA network construction of Toll/Imd signaling pathway

To our knowledge, lncRNAs, known as ceRNAs, can play vital roles in regulating specific target genes by sponging and sequestering miRNAs (Pasquinelli, 2012). Therefore, in order to better construct the ceRNA network based on the lncRNA-miRNA-mRNA axis, all the mRNAs of Toll/Imd signaling pathway were selected from the Section 2.7, and the principle of the selected related lncRNA and miRNA for building the ceRNA network is that the same expression trends of lncRNAs and mRNAs are opposite to the expression of miRNAs, and the *p*-values of lncRNA-miRNA (Pearson correlation coefficients ≥0.6) and lncRNA-miRNA (Pearson correlation coefficients ≥0.6) pairs were less than 0.05. Lastly, all these prediction was imported into Cytoscape software (version 3.9.1) to export the relevant ceRNA regulatory network (Otasek et al., 2019). Besides, the miRNA sequencing and prediction of the selected miRNA-mRNA pairs of Toll/Imd signaling pathway were in agreement with our previous report (data unpublished).

### 2.9 Correlation analysis between RNA transcriptome and 16S rRNA sequencing data

To integrate the transcriptomic and previous 16 S rRNA amplicon sequencing data (data unpublished), Pearson correlation coefficients were calculated for the integrated analysis of the above data. Concretely, the DEMs and DELs of Toll/Imd signaling pathway and the most abundant changes in gut bacterial microbiota from previous 16S rRNA amplicon sequencing were selected to produce correlation heatmaps using R packages (version 2.15.3). The level of statistical significance in this analysis was set at *p < 0.05, **p < 0.01 and ***p < 0.001.

### 2.10 qRT-PCR and statistical analysis

To compare the expression levels between transcriptome sequencing and experimental analysis, fifteen DEMs and ten DELs of the Toll/Imd signaling pathway were used to perform quantitative real-time reverse transcription PCR (qRT-PCR). EF-1α was chosen as an internal control, and other primers were designed with Primer Premier 5 (Supplementary Table S3). Furthermore, the CFX96 real-time PCR detection system (Bio-Rad Laboratories, Hercules, CA) was used to carry out the 20 μL reaction system with PerfectStart Green qPCR SuperMix (Transgen, China) following the manufacturer’s instructions, including 10 μL of PerfectStart™ Green qRT-PCR SuperMix, 0.4 μL of each primer, 2 μL of cDNA template synthesized from total RNA in Section 2.1, and 7.2 μL of nucleotide-free water. The thermal cycling profile consisted of an initial denaturation at 95°C (30 s), followed by 40 cycles of 95°C for 15 s, 60°C for 30 s, and 72°C for 30 s. Finally, relative expression levels were calculated with the 2^−ΔΔCT^ method (Livak and Schmittgen, 2001), and the graphics of expression patterns were drawn by GraphPad Prism 9.0 (GraphPad Software Inc., United States).

## 3 Results

### 3.1 Basic information of sequencing data

In the present research, a total of six transcriptome libraries from the *A. viridicyanea* gut, namely, three control (C) and three antibiotic (A) treatment samples, were sequenced using the MGI2000 platform. Sequencing produced a total of 704,740,664 clean reads from 748,521,486 raw reads after removing low-quality, adaptor and N-containing reads. The mean Q30 and Q20 of each sample were 93.47% and 97.33%, respectively, and the mean GC content of the sequencing data was 47.82% (Supplementary Table S1). Furthermore, all these reads were used for transcript assembly and mapping with StringTie and Hisat 2, respectively, suggesting that different numbers of exons, RNA lengths (mRNA and lncRNA) and transcripts were obtained (Supplementary Figure S1). In addition, the clean reads were submitted to the NCBI SRA (sequence read archive) database under accession number PRJNA946198. All of the above sequencing parameters provided accurate evidence for further analyses.

### 3.2 Identification and classification of mRNAs and lncRNAs

**FIGURE 1:**
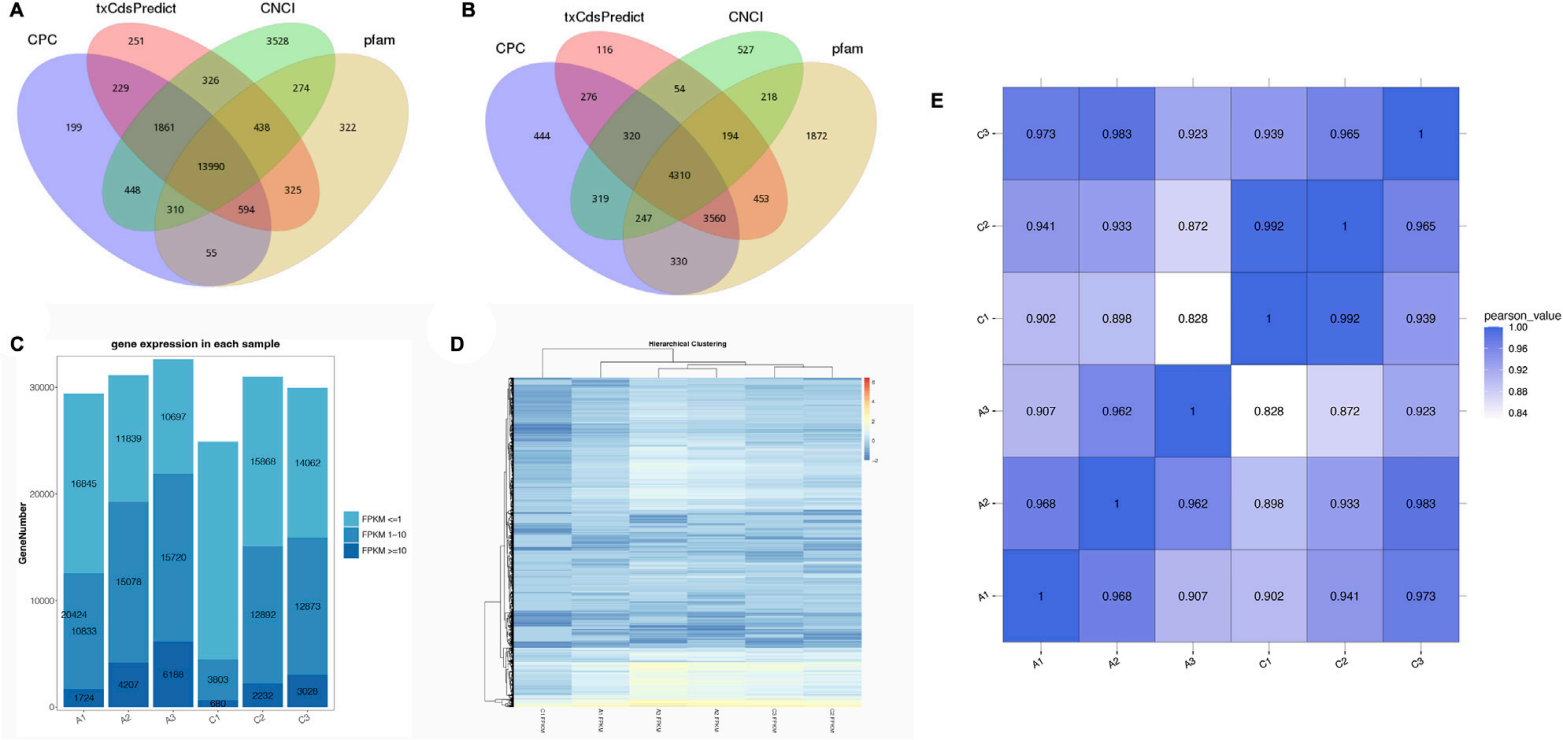
The coding capacity prediction of lncRNAs **(A)** and mRNAs **(B)** are shown in a Venn diagram with CNCI, CPC, txCdsPredict, and pfam. **(C)** The expression profiles of genes from six experimental samples. **(D)** Expression patterns with hierarchical clustering of genes from six experimental groups. **(E)** Correlation heatmap of RNA sequencing among the six transcriptome libraries.

Coding potential methods, including CPC, CNCI, Pfam, and txCdsPredict, were applied to screen the transcripts of mRNAs or lncRNAs. A total of 17,193 lncRNAs and 26,361 mRNAs (17,730 known mRNAs and 8,631 novel mRNAs) were obtained based on the prediction (Figure 1A, B). The lengths of lncRNAs and mRNAs, and the number of exons and transcripts are shown in Supplementary Figure S2.

### 3.3 Expression levels of lncRNAs and mRNAs

At first, the FPKM values were calculated to estimate the expression patterns of all genes. In the C1 to C3 groups, the FPKM values were mainly less than 1, while the FPKM values were mainly between 1 and 10 in the A2 and A3 groups, except for the A1 group (Figure 1C). In our study, a heatmap was used to visualize all differentially expressed RNAs (Figure 1D). In addition, the correlation relationships (*R* ^2^ values) represented the robustness of biological replicates and the reliability of RNA-seq data among the six groups (Figure 1E).

**FIGURE 2:**
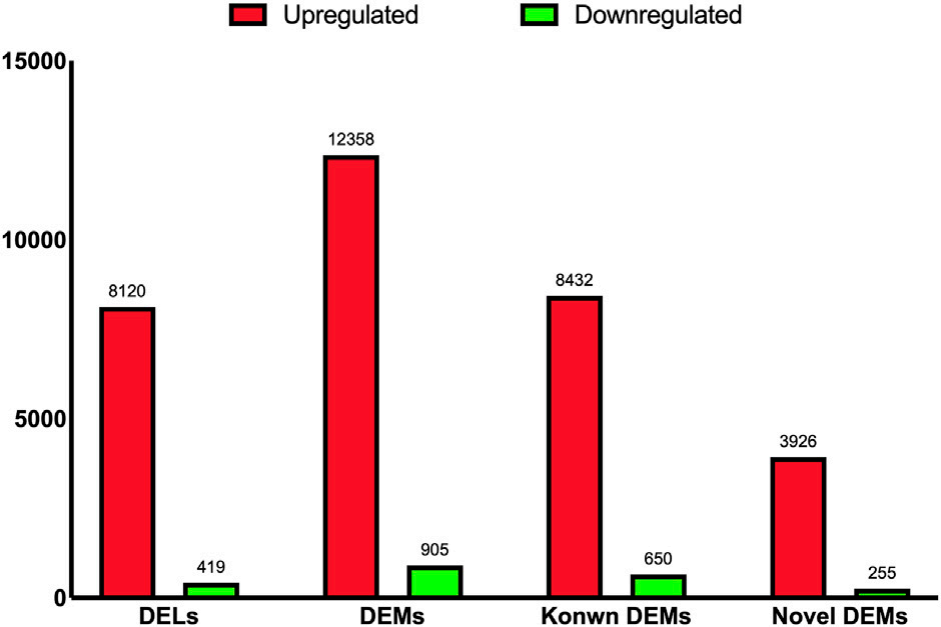
The bar chart shown the numbers of differential expressed lncRNAs, mRNAs, known mRNAs, and novel mRNAs.

As shown in Figure 2, a total of 8,539 DELs were identified, including 8,120 upregulated and 419 downregulated DELs between the two experimental groups. In addition, 12,358 and 905 DEMs were upregulated and downregulated, respectively, from a total of 13,263 DEMs between the A and C groups. Of these DEMs, 8,432 and 3,926 were known and novel upregulated DEMs, and 650 and 255 were known and novel downregulated DEMs, respectively (Figure 2).

### 3.4 Target prediction of lncRNAs

For target prediction of lncRNAs, lncRNA-mRNA interactions were screened based on the results of coexpression and colocalization. The results indicated that a total of 6,409 lncRNA-mRNA interaction pairs were cis/trans-acting from 6,035 lncRNAs targeting 6,421 mRNAs. In addition, a total of 4,086 lncRNA-mRNA pairs overlapped from 3,097 lncRNAs targeting 1985 mRNAs (Supplementary Figure S3). In addition, all DELs target a total of 6,425 DEMs in this research.

### 3.5 Functional enrichment analysis for mRNAs, DELs and target genes

**FIGURE 3:**
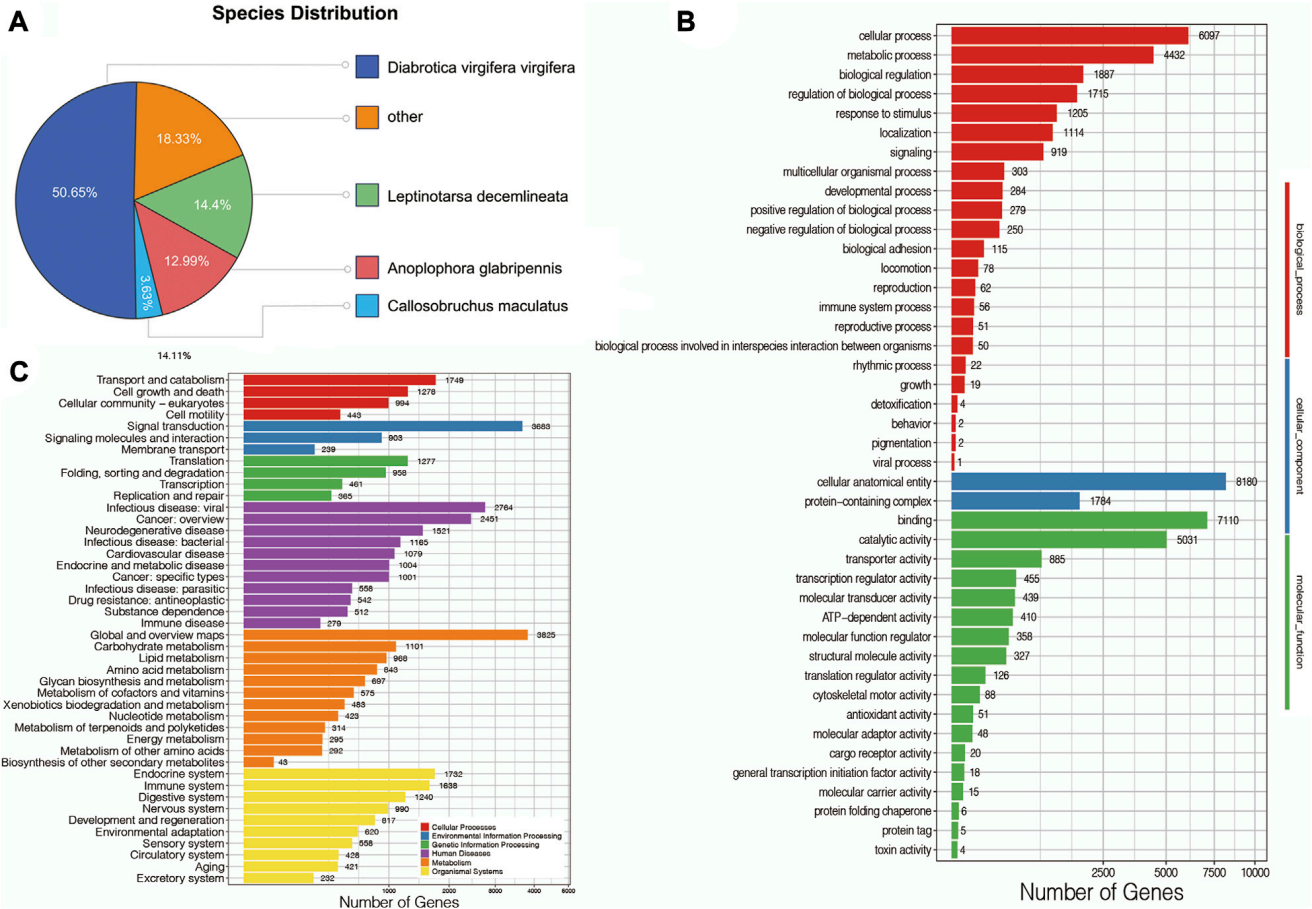
**(A)** Species distribution of *A. viridicyanea* mRNAs. **(B)** GO annotation of all mRNAs. *X*-axis: the number of mRNAs annotated in the GO groups, *Y*-axis: three major functional categories of GO terms including biological process (Red), cellular component (Blue), and molecular function (Green). **(C)** KEGG classification of all mRNAs. *X*-axis: the number of mRNAs annotated in the KEGG database, *Y*-axis: six major categories of KEGG pathway including cellular processes (Red), environmental information processing (Blue), genetic information processing (Green), human diseases (Purple), metabolism (Orange), and organismal systems (Yellow).

First, to obtain the basic annotation information of assembled mRNAs, all of them were compared against several databases. In detail, 25,594 mRNAs (97.09%) were annotated in at least one database, and 25,153 (95.42%), 13,072 (49.59%), 18,188 (69%), 21,278 (80.72%), 16,660 (63.20%) and 13,441 (50.99%) mRNAs were annotated against the Nr, Nt, SwissProt, KEGG, KOG, Pfam, and GO databases, respectively. Based on the Nr annotation, this transcriptome was mostly comparable to *D. virgifera virgifera* (50.65%), followed by *Leptinotarsa decemlineata* (14.4%), *Anoplophora glabripennis* (12.99%) and *Callosobruchus maculatus* (3.63%) (Figure 3A). Next, the GO database was used to comprehensively and widely investigate the basic functions of mRNAs, suggesting that a total of 13,441 mRNAs were classified into three GO categories, including biological process, cellular component, and molecular function (Figure 3B), such as 6,097 mRNAs in cellular process, 8,180 mRNAs in cellular anatomical entity, and 7,110 mRNAs in binding. Furthermore, the KEGG database was also used to systematically analyze gene functions. As shown in Figure 3A, C, total number of 18,188 mRNAs were assigned to six main categories of KEGG metabolic pathways: cellular processes, environmental information processing, genetic information processing, human diseases, metabolism, and organismal systems, especially 1,638 mRNAs in the immune system.

**FIGURE 4:**
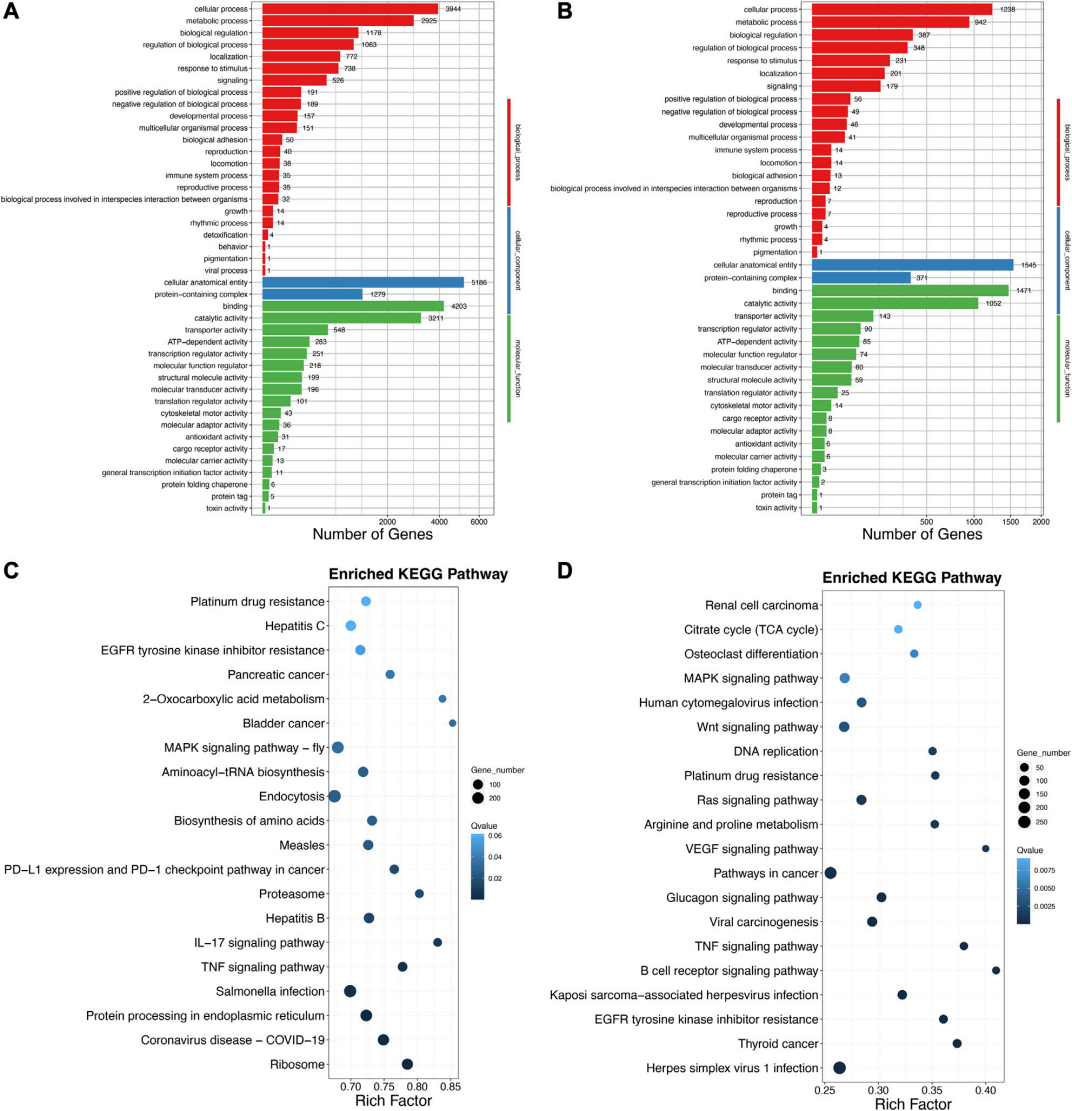
GO classification and KEGG annotation of the DELs **(A–C)** and their target genes **(B–D)**. *X*-axis of Figure **(A**, **B)** the number of mRNAs annotated in the GO database; and *Y*-axis: three major functional categories of GO terms including biological process (Red), cellular component (Blue), and molecular function (Green). *X*-axis of Figure **(C**, **D)** the percentages of mRNAs belong to the corresponding pathway, and *Y*-axis: the top 20 pathways. The sizes of bubble represent the number of genes in the corresponding pathway, and the colors of the bubble represent the enrichment q-value of the corresponding pathway.

Second, to ascertain the functional details of the DELs and target genes, GO and KEGG enrichment analyses were carried out. Based on the above GO categories, most DELs were distributed in cellular process, cellular anatomical entity, binding, catalytic activity, and so forth (Figure 4A). Likewise, 5,186 target genes were found in cellular anatomical entity, followed by 4,203 target genes in binding, 3,944 target genes in cellular processes, and 3,211 target genes in catalytic activity (Figure 4B). Furthermore, the top 20 significantly enriched pathways of DELs and target genes using KEGG enrichment indicated some immune signaling pathways, including the MAPK signaling pathway, TNF signaling pathway, B-cell receptor signaling pathway, IL-17 signaling pathway, and MAPK signaling pathway-fly (Figure 4C, D). In addition, we also summarized the various immune signaling pathways in the *A. viridicyanea* gut with different numbers of DELs and DEMs (Table 1). These collected data suggested that lncRNAs may contribute to the regulation of mRNA expression levels related to immune signaling pathways, which could help us to illustrate the immune responses of beetles under pathogen stimulation in the future.

**TABLE 1:** Summary of DELs and DEMs in immune-related pathways.

Pathway ID	Pathway name	DEL numbers	DEL ratio	DEM numbers	DEM ratio
ko04624	Toll/Imd signaling pathway	44	0.00515283	117	0.00882153
ko04620	Toll-like receptor signaling pathway	33	0.00386462	80	0.00603182
ko04621	NOD-like receptor signaling pathway	47	0.00550416	120	0.00904773
ko04622	RIG-I-like receptor signaling pathway	34	0.00398173	78	0.00588102
ko04630	JAK/STAT signaling pathway	32	0.00374751	63	0.00475006
ko04064	NF-κB signaling pathway	40	0.00468439	107	0.00806756
ko04151	PI3K-Akt signaling pathway	89	0.01042277	223	0.01681369
ko04657	IL-17 signaling pathway	22	0.00257641	49	0.00369449
ko04010	MAPK signaling pathway - fly	91	0.01065699	232	0.01749227
ko04142	Lysosome	88	0.01030566	286	0.02156375
ko04668	TNF signaling pathway	41	0.0048015	84	0.00633341
ko04625	C-type lectin receptor signaling pathway	48	0.00562127	98	0.00738898
ko04350	TGF-beta signaling pathway	42	0.00491861	90	0.0067858

### 3.6 Identification and characterization of Toll/Imd pathway genes

**FIGURE 5:**
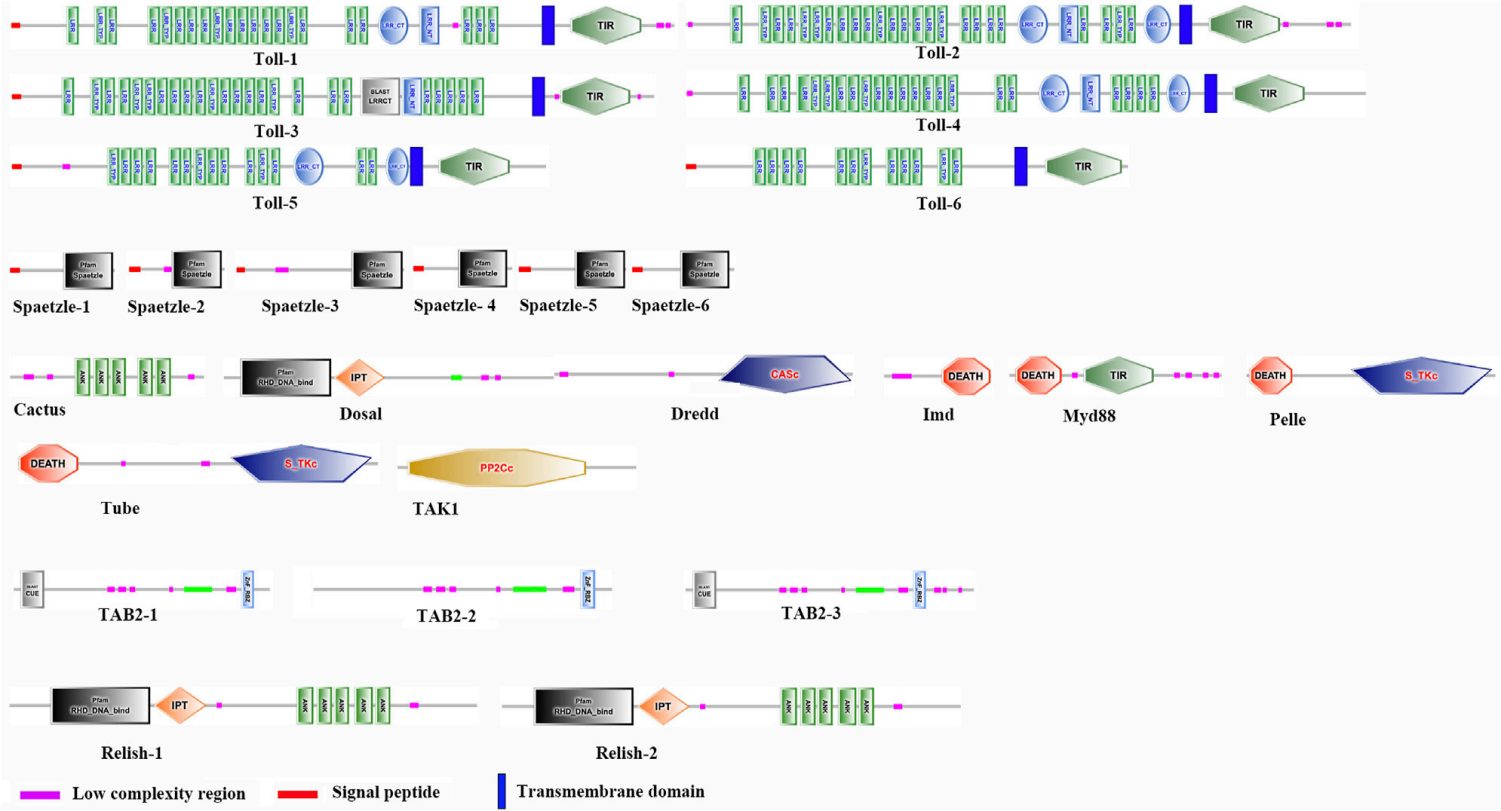
Domain organizations of Toll/Imd signaling pathway proteins in *A. viridicyanea* using SMART prediction. Complex domains are marked with different shapes and colors.

In order to comprehensively understand one essential immune pathway in insects, we further focused on investigation of Toll/Imd pathway genes and their ceRNA network in *A. viridicyanea* based on sequencing data. Our observations suggested that six full-length cDNA sequences of *Toll* family genes were identified based on annotation information and online tools. The cDNA and protein characterization of all *Tolls* are shown in Table 2, and all sequences have been deposited in the NCBI database under accession numbers from OQ819300 to OQ819306. Concretely, all ORF lengths ranged from 3,972bp to 2,571 bp and encoded polypeptides 1,323 to 856 amino acids (aa) with molecular weights (MWs) of 149.45–98.26 kDa and theoretical isoelectric points (PIs) of 5.66–8.54, respectively. All Tolls contain different numbers of extracellular serial leucine-rich repeat (LRR) motifs, a transmembrane domain, and an intracellular cytoplasmic Toll/IL1 receptor (TIR) domain (Figure 5). Moreover, based on the classification of invertebrate *Tolls* described by Gerdol et al. (2017), six *A. viridicyanea Tolls* were divided into three subfamilies: four P-type *TLRs* (*Toll-1* to *Toll-4*), one sPP-type *TLR* (*Toll-5*), and one sP-type *TLR* (*Toll-6*), whose subcellular location is the plasma membrane (Table 2).

**TABLE 2:** The cDNA and protein characterization of *Toll* family genes.

Gene name	ORF length (bp)	Amino acids length (aa)	Molecular weight (kDa)	Theoretical pI	Signal peptide motif (aa)	LRR motif (aa)	Transmembrane motif (aa)	TIR motif (aa)	Subcellular localization	NCBI accession number
Toll-1	3,855	1,284	147.03	5.77	1–20	108–131, 162–185, 186–205, 265–288, 289–312, 314–337, 338–360, 364–387, 388–411, 412–435, 436–459, 460–479, 483–506, 507–530, 531–554, 555–574, 647–668, 671–691, 711–769, 791–829, 872–893, 896–917, 920–943	1,029–1,051	1,083–1,220	Plasma membrane	OQ819300
Toll-2	3,864	1,287	146.34	5.69	1–17	86–109, 141–164, 165–190, 193–212, 218–241, 242–265, 266–285, 290–315, 316–339, 340–363, 364–387, 388–411, 412–431, 435–461, 459–482, 483–506, 530–552, 553–571, 581–596, 599–618, 642–700, 722–760, 759–778, 803–824, 827–850, 851–870, 886–938	956–978	1,012–1,149	Plasma membrane	OQ819301
Toll-3	3,735	1,244	141.20	5.99	1–19	98–121, 153–176, 177–200, 207–230, 231–254, 255–278, 279–302, 303–328, 329–350, 353–376, 377–400, 401–424, 425–447, 448–471, 472–495, 496–519, 543–565, 612–633, 637–659, 756–795, 796–813, 815–837, 838–859, 862–883, 886–914	1,009–1,031	1,063–1,197	Plasma membrane	OQ819302
Toll-4	3,972	1,323	149.45	5.66	1–15	100–123, 154–180, 178–201, 214–246, 238–261, 262–285, 286–312, 313–335, 336–359, 360–383, 384–407, 408–431, 432–453, 455–481, 479–502, 503–526, 597–618, 619–640, 683–741, 763–801, 821–843, 844–865, 868–889, 892–915, 930–975	1,004–1,026	1,058–1,196	Plasma membrane	OQ819303
Toll-5	3,108	1,035	119.36	5.84	1–20	186–208, 209–232, 233–254, 257–280, 305–328, 329–348, 353–376, 377–400, 401–421, 452–471, 474–497, 500–520, 546–604, 667–688, 687–707, 725–769	773–795	827–964	Plasma membrane	OQ819304
Toll-6	2,571	856	98.26	8.54	1–21	131–155, 156–179, 184–207, 211–233, 290–312, 313–336, 337–360, 388–411, 412–435, 436–458, 488–511, 512–535	638–660	696–844	Plasma membrane	OQ819306

Furthermore, to explore the signal members of the Toll/Imd pathway in *A. viridicyanea*, we also selected a total of nineteen extracellular and intracellular genes according to transcriptome sequencing. As shown in Table 3, six *Spaetzle*, three *TAB2*, two *Relish*, and one *Myd88*, *Pelle*, *Tube*, *Cactus*, *Dorsal*, *Imd*, *Dredd* and *TAK1* genes were screened with complete coding DNA sequence (CDS) regions. Furthermore, we also characterized the above protein sequences in *A. viridicyanea*, including the length of the protein, molecular weight, isoelectronic point and functional domain, indicating that they possess typical domains for signal transduction or binding, such as the DEATH, Spaetzle, ANK, and RHD_DNA_bind domains. All these data showed that information on all Toll/Imd pathway genes with typical functional domains could expand our knowledge for comparative immunology between *A. viridicyanea* and other insects.

**TABLE 3:** The cDNA and protein characterization of Toll/Imd pathway genes.

Gene name	ORF length (bp)	Amino acids length (aa)	Molecular weight (kDa)	Theoretical pI	Functional domain and motif (aa)	NCBI accession number
Spaetzle-1	612	203	23.62	7.99	Sp: 1–20; Spaetzle: 104–199	OQ834310
Spaetzle-2	561	186	21.09	7.46	Sp: 1–23; Spaetzle: 84–180	OQ834311
Spaetzle-3	975	324	37.82	9.17	Sp: 1–17; Spaetzle: 223–321	OQ834312
Spaetzle-4	579	192	22.30	9.32	Sp: 1–21; Spaetzle: 88–182	OQ834313
Spaetzle-5	624	207	23.98	5.51	Sp: 1–24; Spaetzle: 109–205	OQ834314
Spaetzle-6	597	198	22.88	9.32	Sp: 1–21; Spaetzle: 94–188	OQ834315
Myd88	1,236	411	47.17	6.02	DEATH: 12–101; TIR: 144–279	OQ834300
Pelle	1,449	482	55.31	8.53	DEATH: 6–89; S_TKc: 203–475	OQ834301
Tube	2091	696	77.87	4.86	DEATH: 1–116; S_TKc: 411–684	OQ834302
Cactus	1,125	374	41.90	4.83	ANK: 125–154, 161–190, 194–223, 246–275, 280–310	OQ834303
Relish-1	2,718	905	103.25	5.51	RHD_DNA_bind: 80–272; IPT: 279–381; ANK: 556–588, 595–624, 628–657, 668–703, 708–737	OQ834304
Relish-2	2,664	887	101.13	5.58	RHD_DNA_bind: 62–254; IPT: 261–363; ANK: 538–570, 577–606, 610–639, 650–685, 690–719	OQ834305
Dorsal	2,235	744	83.55	8.68	RHD_DNA_bind: 62–254; IPT: 261–363	OQ834306
Imd	621	207	28.84	6.54	DEATH: 111–205	OQ834307
Dredd	1734	577	66.16	6.10	CASc: 318–574	OQ834308
TAK1	1,386	461	51.55	4.98	PP2Cc: 20–364	OQ834309
TAB2-1	1,488	495	55.99	8.72	CUE: 14–59; ZnF_RBZ: 440–464	OQ834316
TAB2-2	1,479	492	55.64	8.81	ZnF_RBZ: 440–464	OQ834317
TAB2-3	1,674	557	62.57	8.77	CUE: 14–59; ZnF_RBZ: 440–464	OQ834318

**FIGURE 6:**
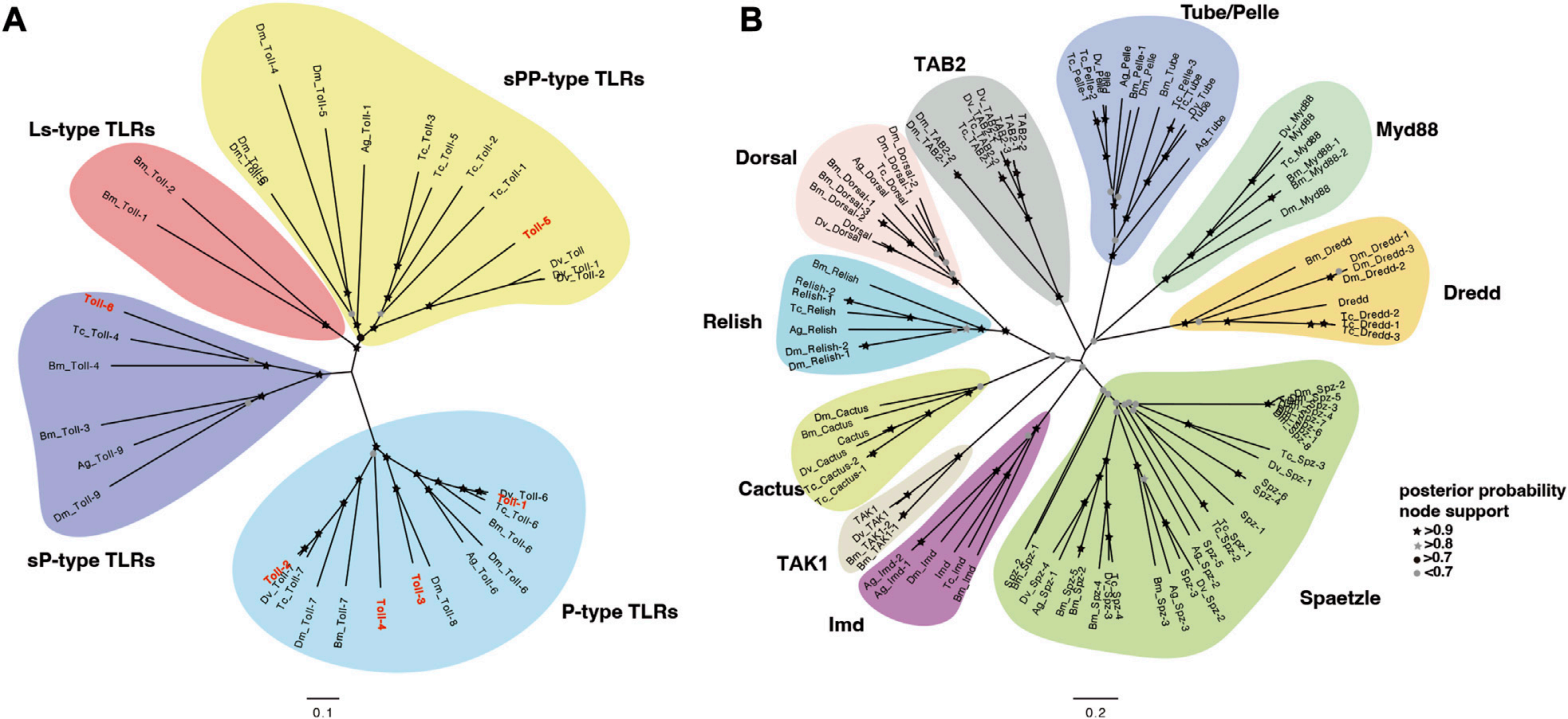
Phylogenetic analysis of Toll/Imd signaling pathway genes in *A. viridicyanea* and other insects based on the neighbor-joining method using the program MEGAX with 1,000 bootstrap values. **(A)** Toll family genes, and **(B)** extracellular and intracellular factors. All accession numbers of amino acid sequences used for phylogenetic tree construction are summarized in Supplementary Table S2, and nodes with posterior probability supports are shown by black or grey colors of circles and pentacles.

To evaluate the evolutionary relationships among the above family genes, the protein sequences from five insects were aligned to construct neighbor-joining trees (Supplementary Table S2). In line with our classification, the phylogeny result based on a phylogenetic tree with NJ method implied that the *A. viridicyanea Tolls* were well clustered together with corresponding subfamilies in insects, including P-type *Tolls*, sPP-type *Tolls* and sP-type *Tolls* (Figure 6A). All the pathway genes were also well clustered into each gene family (Figure 6B), which supports their orthologous relationships. Next, the immune recognition and signal transduction of all these genes will be validated in the *A. viridicyanea* gut.

**FIGURE 7:**
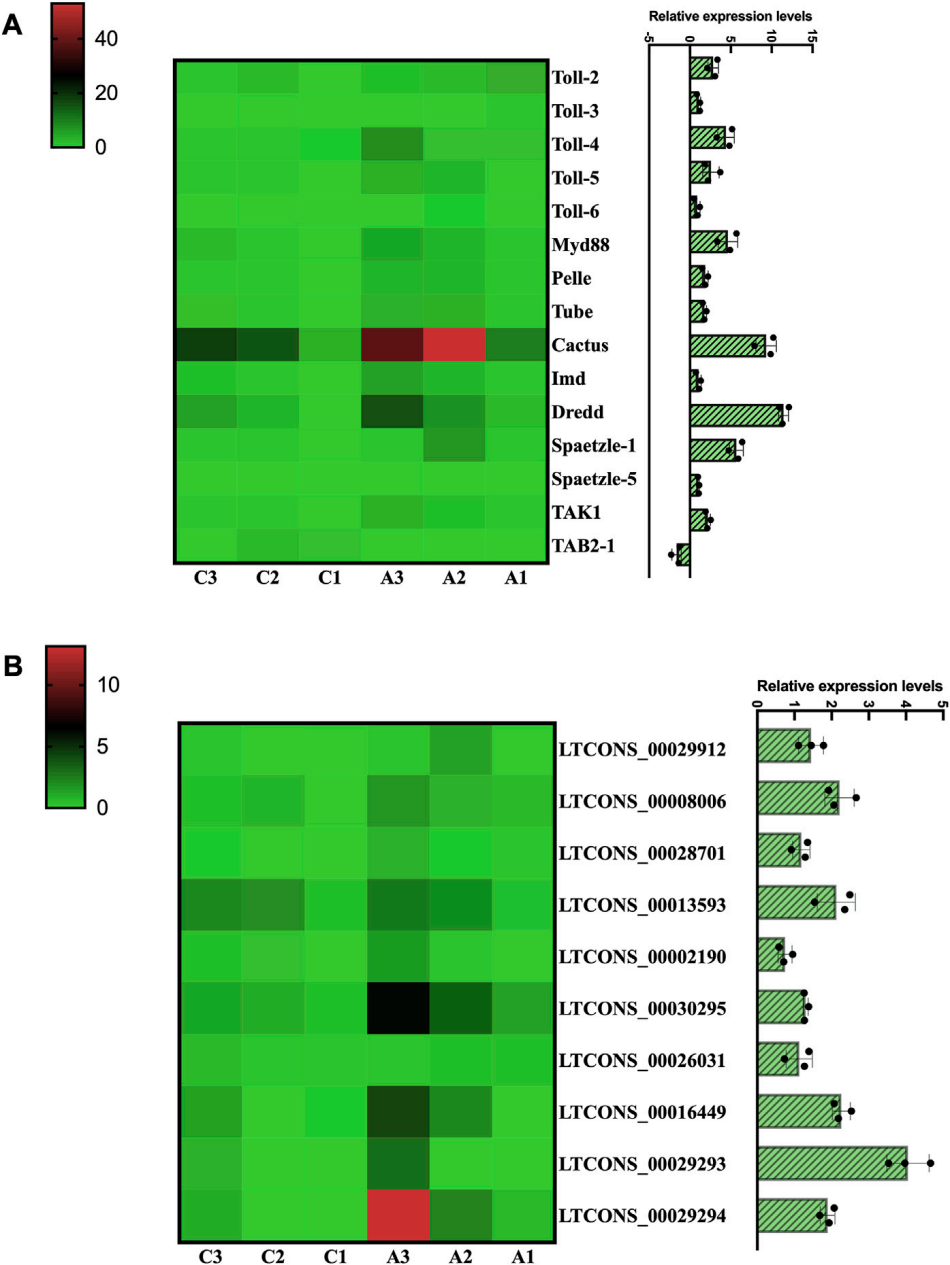
Transcription analysis of DEMs and DELs involved in the Toll/Imd signaling pathway. Heatmap of DEMs **(A)** and DELs **(B)** associated with the Toll/Imd signaling pathway. Heatmap colors correspond to the scale values of FPKM. The histograms represent the expression of DEMs **(A)** and DELs **(B)** by qRT-PCR analysis.

Finally, to better understand the expression changes of Toll/Imd signaling pathway genes under antibiotic treatment, we calculated and summarized the profiles of DEMs and DELs of Toll/Imd pathway according to RNA-seq data. The results suggested that we found differential expression patterns of five *Tolls*, two *Spaetzles* and some pathway genes between the C and A groups. Most of these genes were significantly upregulated, while *TAB2-1* expression significantly decreased during antibiotic rearing. Notably, the expression levels of all NF-κB-related genes were not significantly changed in this study (Figure 7A). In addition, the expression trends by qRT‒PCR were consistent with the differential expression of RNA sequencing, which verified the reliability of our sequencing data.

### 3.7 Establishment of a lncRNA-mediated ceRNA network of the Toll/Imd pathway

**FIGURE 8:**
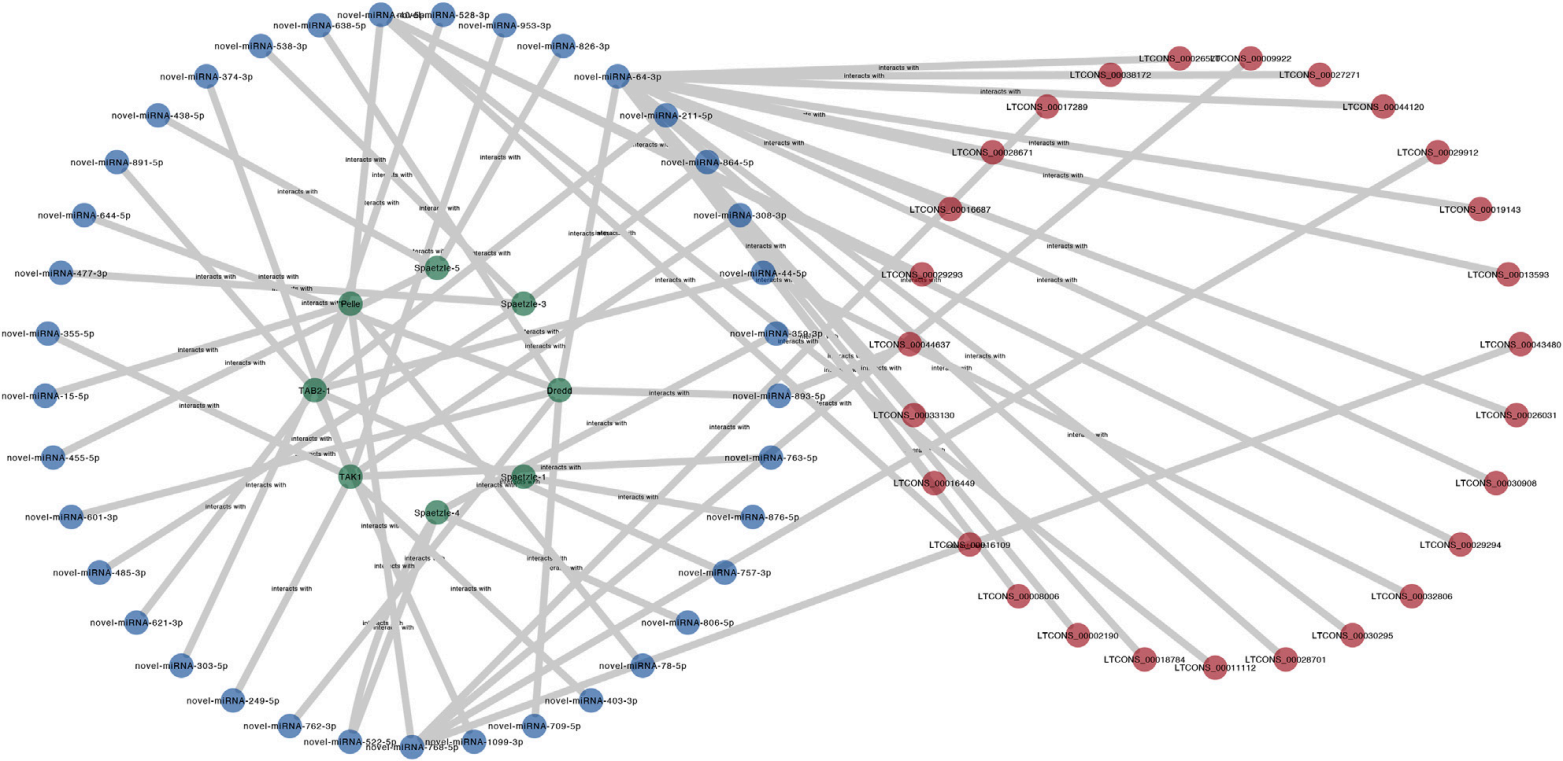
The predicted lncRNA-miRNA-mRNA network of the Toll/Imd signaling pathway. LncRNA, miRNA and mRNA are represented by red, blue and green ellipses, respectively.

In this research, to investigate the miRNA sponge capacity of lncRNAs, eight Toll/Imd pathway genes, including *Pelle*, *Dredd*, *Spaetzle-1*, *Spaetzle-3*, *Spaetzle-4*, *Spaetzle-5*, *TAK1* and *TAB2-1*, and their related miRNAs and lncRNAs were predicted and selected to construct a ceRNA network (Figure 8). In total, 76 miRNA‒mRNA pairs and 54 lncRNA‒miRNA pairs were obtained by target prediction. Of these lncRNAs and mRNAs, 10 DELs were involved in the regulation of 6 DEMs in this predicted lncRNA-mediated ceRNA network. In detail, *Dredd*, *TAK1* and *TAB2-1* are regulated by 7 miRNAs, and five miRNAs contribute to target *Pelle*. Meanwhile, 12 miRNAs were found to combine *Spaetzle* family genes. In addition, the novel miRNA-64-3p targets the majority of lncRNAs in the lncRNA-related ceRNA network of Toll/Imd signaling pathway. As shown in Figure 7B, a heatmap was used to display all above DELs with increasing expression between control and antibiotic groups, which brings together transcriptome sequencing and qRT-PCR validation with similar expression patterns.

### 3.8 Correlation analysis between DELs or DEMs of the Toll/Imd pathway and gut bacterial microbiota

**FIGURE 9:**
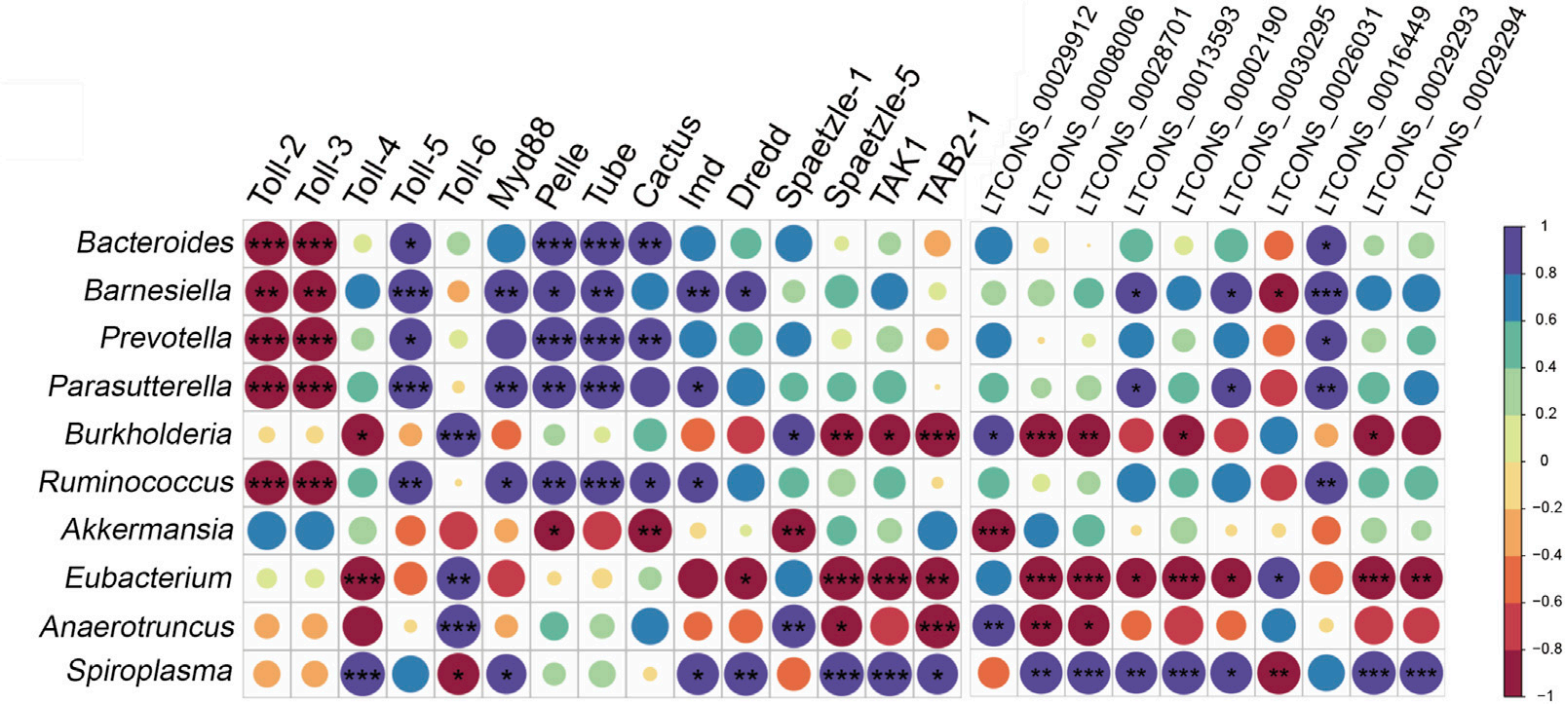
Integrated analysis between DEMs or DELs and gut bacterial microbiota using Pearson correlation coefficients. Correlation coefficients are shown in different colors, which represent different degrees of positive or negative correlation. Ellipses sizes are proportional to correlation value and correlation significance is performed by *p < 0.05, **p < 0.01 and ***p < 0.001.

We hypothesized that DELs and DEMs of the Toll/Imd pathway might be involved in response to alterations in the gut bacterial microbiota. Therefore, we detected their association relationships with Pearson correlation coefficients under antibiotic treatment. The results shown in Figure 9 suggest that, after antibiotic treatment, all selected changes in the gut bacterial microbiota were significantly positively or negatively correlated with different numbers of DEMs in the Toll/Imd signaling pathway. In detail, *Toll-2* and *Toll-3* changes were significantly positively affected by alterations in *Bacteroides*, *Barnesiella*, *Prevotella*, *Parasutterella* and *Ruminococcus*, whereas they significantly negatively influenced *Toll-5* expression. In contrast, changes in *Burkholderia*, *Eubacterium* and *Spiroplasma* were only significantly correlated with *Toll-4* and *Toll-6* expression changes. Furthermore, changes in some gut bacterial microbiota, such as *Bacteroides*, *Barnesiella*, *Prevotella*, *Parasutterella* and *Ruminococcus*, were mainly significantly negatively involved in influencing some downstream factors, such as *Myd88*, *Tube*, *Pelle*, *Imd*, *Dredd* and *Cactus*, but *Akkermansia* alteration played the opposite role in affecting the expression of *Pelle* and *Cactus*. In addition, alterations in *Eubacterium* and *Spiroplasma* significantly positively or negatively affected the majority of the expression of DELs related to the Toll/Imd signaling pathway. However, *Bacteroides*, *Prevotella*, *Ruminococcus* and *Akkermansia* changes were only significantly positively or negatively associated with one DEL (Figure 9).

## 4 Discussion

*A. viridicyanea* serves as a good material for investigating the adaptive evolution between insects and host plants. However, there have been few studies on the innate immunity of *A. viridicyanea* due to the lack of an adaptive immune system, which mainly depends on nonspecific immune responses, such as the activation of immune signaling pathways and production of antimicrobial peptides (Lin et al., 2022; Wei et al., 2022). LncRNAs, incapable of translation into proteins, are significant regulators that regulate gene expression and are involved in various physiological and immunological functions at the transcriptional and posttranscriptional levels, such as the lncRNA-miRNA-mRNA axis based on the competitive endogenous RNA hypothesis (Zhang et al., 2020; Lin et al., 2022). In the last decade, accumulating evidence has confirmed the important roles of lncRNAs in the regulation of innate immunity in insects (Valanne et al., 2019; Zhang et al., 2020; Zhang et al., 2021a; Moure et al., 2022). In this study, we performed transcriptome analysis to detect the characterization and expression profiles of lncRNAs and mRNAs in the *A. viridicyanea* gut during normal rearing and antibiotic feeding, constructed a lncRNA-mediated ceRNA network of the Toll/Imd signaling pathway, and finally analyzed the correlation between their related DEMs or DELs and changes in the gut bacterial microbiota.

To date, a growing number of lncRNAs have been reported with the development of transcriptome sequencing from various tissues or cells in insects, such as the fat body, silk gland, gonad, and cell lines (Wu et al., 2016b; Lin et al., 2022). We used the gut tissues of *A. viridicyanea* to carry out transcriptome sequencing in this report, as it is an important digestive and immune tissue in insects. Our study indicated that we obtained a large number of raw data and clean reads, and among these reads, a total of 17,193 lncRNAs and 26,361 mRNAs were identified, which was more than some previous reports (Wu et al., 2016b; Zhang et al., 2020). In addition, the average exon numbers and RNA lengths of all selected lncRNAs were less and shorter than those of all identified mRNAs in the *A. viridicyanea* gut, suggesting that lncRNAs possess sequence conservation for facilitating their binding function.

Furthermore, differential expression analysis of lncRNAs and mRNAs was conducted to identify DELs and DEMs and obtain their functional annotation using different databases. A total of 8,539 lncRNAs were differentially expressed, which consisted of 8,120 lncRNAs with increasing expression and 419 lncRNAs with decreasing expression after antibiotic feeding, indicating the potential participation of these lncRNAs in various functions in the *A. viridicyanea* gut. We further detected 12,358 upregulated and 905 downregulated DEMs. Consistent with previous research, this finding also implied that lncRNAs are expressed at relatively lower levels than mRNAs in the *A. viridicyanea* gut due to their high tissue specificity for exerting specific biological functions (Wu et al., 2016b). Herein, among all these DELs and DEMs, our results illustrated that they were strongly enriched in immune signaling pathways based on GO and KEGG analysis. In detail, the lysosome, MAPK signaling pathway–fly and PI3K-Akt signaling pathway were enriched in the majority of DELs and DEMs. PRR-related pathways, including the NOD-like receptor, Toll-like receptor, C-type lectin receptor, Toll/Imd and RIG-I-like receptor signaling pathways, were also selected, which may play pivotal roles in pathogenic recognition or complementary function during changes in the gut bacterial microbiota of *A. viridicyanea* (Takeuchi and Akira, 2010). The JAK/STAT signaling pathway in insects is similar to type I interferon signaling in mammals, which induces the transcription of genes to prevent viral replication (Stark and Darnell, 2012). In addition, the interleukin-17 (IL-17) family includes ancient proinflammatory cytokines that combine their specific receptors (IL-17 receptor, IL-17R) to protect the host against pathogens (Cua and Tato, 2010). Collectively, our data show that immune DELs and DEMs might be involved in response to antibiotic treatment in the *A. viridicyanea* gut.

Toll receptors are important PRRs and type I transmembrane proteins, containing extracellular LRR domain, transmembrane domain, and intracellular TIR domain for recognizing conserved PAMPs and initiating downstream signaling pathways from invertebrates to vertebrates (Medzhitov, 2001; Kawai and Akira, 2010). In invertebrates, multiple cysteine cluster TLRs (mccTLRs) and single cysteine cluster TLRs (sccTLRs) were found and classified based on LRR ectodomain arrangements, but only sccTLRs exist in vertebrate animals (Gerdol et al., 2017). In this study, we selected six *Toll* family genes from transcriptome sequencing data, which are less than the number of *Drosophila* and silkworm *Tolls* (Zhang et al., 2021b). Of these *A. viridicyanea Tolls*, three subfamilies, including four P-type *TLRs* (*Toll-1* to *Toll-4*) resembling *Drosophila Tolls*, one sPP- type *TLR* (*Toll-5*) with the same LRR arrangement but shorter than P-type *TLRs*, and one sP- type *TLR* (*Toll-6*) shorter than vertebrate *TLRs*, were grouped, suggesting that the number and diversity of insect *Tolls* are less than those in mollusk and echinozoa due to their expansion in most deuterostomes and Lophotrochozoa (Satake and Sekiguchi, 2012; Zhang et al., 2015; Gerdol et al., 2017). Furthermore, *Imd* is another essential immune defense factor in the *Drosophila* gut that has been implicated in protection against bacterial pathogens (Lemaitre et al., 1995). Here, we selected an *Imd* gene, translating a DEATH domain to facilitate signal transduction, in *A. viridicyanea*.

In *Drosophila*, the Toll pathway was discovered to be contributed to fungal and bacterial infections and activation by *Spaetzles* and *PGRPs*. For regulation of the innate immune system in *Drosophila*, PGRP-SA cooperates with PGRP-SD in the hemolymph and is required for the activation of Toll pathway by binding to Lys-type PGN (peptidoglycan), and PGRP-LC and PGRP-LE recognize the DAP-type PGN of gram-negative bacteria to activate the Imd pathway (Zhang et al., 2019). Next, the recognition of PAMPs results in the Spaetzle activation, and then causes the dimerization of Tolls, which combine with the intracellular adaptor Myd88 to recruit Tube and Pelle, participate in the phosphorylation and degradation of Cactus, and finally release Dorsal or Dif to induce the expression of AMPs (Valanne et al., 2022). Furthermore, to initiate the Imd signaling pathway, the receptors PGRP-LE and PGRP-LC detect bacterial ligands to promote the interaction between Imd and Fadd, leading to caspase Dredd activation, and then, Relish is released into the nucleus to produce AMPs (Silverman et al., 2003). In our study, we obtained a total of six extracellular *Spaetzle* genes, whose numbers are similar to those in *D. melanogaster* (Parker et al., 2001), and different numbers of intracellular signal factors in the Toll/Imd pathway, except for the *Fadd* gene, suggesting that a classical Toll/Imd pathway may exist in the *A. viridicyanea* gut. Next, more evidence on molecular and comparative immunology between model insects and *A. viridicyanea* will be applied to tackle the questions of immune signal transduction and function of the Toll/Imd pathway in *A. viridicyanea* under bacterial pathogen infection.

LncRNA regulation involves a complex matrix of coding and noncoding RNAs at the transcriptional or posttranscriptional level and has been demonstrated to be related to the modulation of the expression of immune- or metabolism-related genes (Li et al., 2018). For instance, *Drosophila* lncRNA-CR33942 and lncRNA-CR46018 interact with the transcription factors *Dif* and *Dorsal* to promote the Toll pathway (Zhou et al., 2021b; Zhou et al., 2022a; Zhou et al., 2022b). To date, an increasing number of investigations have shown that gene expression can be controlled through lncRNA-mediated ceRNAs with miRNA binding in animals. Specifically, in the ceRNA network, miRNAs interact with lncRNAs, serving as precursors or pre-miRNA enhancers, by miRNA-binding sites, leading to the regulation of gene expression (Pilyugin and Irminger-Finger, 2014; Jiang et al., 2017). In this study, eight mRNAs of the Toll/Imd signaling pathway were targeted by thirty-eight miRNAs interacting with twenty-seven lncRNAs in the *A. viridicyanea* gut under antibiotic treatment, while *Toll* family genes were not controlled by miRNAs and lncRNAs in our research. Of these lncRNAs and mRNAs, ten DELs contributed to regulate six DEMs of the Toll/Imd signaling pathway by combining with various miRNAs. For example, two DELs, LTCONS_00044637 and LTCONS_00032806, competitively bind novel-miRNA-893-5p and novel-miRNA-44-5p to regulate the significantly differential expression of *Dredd* and *TAB2-1*, respectively. Moreover, the majority of DELs were involved in the expression of essential signal factors, *Pelle* and *Dredd*, by competing with the target novel-miRNA-40-5p, novel-miRNA-768-5p, novel-miRNA-893-5p and novel-miRNA-64-3p.

Previous studies on the relationships between the Toll/Imd pathway and commensal bacteria illustrated that the Imd pathway of *Drosophila* maintains homeostasis of the barrier epithelium with gut bacterial symbionts, which may also be involved in Toll activities during viral infections in the fly gut (Chow and Kagan, 2018). Due to an imbalance in gut microbiota composition influencing host fitness, we determined whether changes in the gut bacterial microbiota affected the lncRNA and mRNA expression of the Toll/Imd pathway in *A. viridicyanea* during antibiotic treatment. On the basis of the above observations, we found that disturbances in the gut bacterial microbiota can influence the expression of some lncRNAs and mRNAs in the Toll/Imd pathway in the *A. viridicyanea* gut, demonstrating that host innate immunity through lncRNA-mediated ceRNAs may play essential roles in response to changes in the gut bacterial microbiota of *A. viridicyanea*. Likewise, there are growing numbers of reports on the gut bacterial microbiota affecting the expression of immune genes in insects. For example, the depression of the *thanatin* gene, an antimicrobial peptide, in *Riptortus pedestris* significantly improved the population of *Burkholderia* gut symbionts during *Escherichia coli* injection (Lee et al., 2022). Additionally, the gut bacterial microbiota of *Apis mellifera* and *Spodoptera frugiperda* can maintain AMP expression in gut tissues in response to future adverse environments (Kwong et al., 2017; Zheng et al., 2023). In red palm weevil (RPW), *Rhynchophorus ferrugineus*, the effects of the Toll signaling pathway on gut bacterial composition are highly limited and nonspecific under *Spaetzle* knockdown (Muhammad et al., 2020). Taken together, further studies will be conducted to confirm the lncRNA-related ceRNA regulatory network of the Toll/Imd pathway and elucidate their roles in maintaining gut homeostasis in *A. viridicyanea*.

## 5 Conclusion

In conclusion, we first identified and analyzed lncRNAs and mRNAs in normal and antibiotic-fed *A. viridicyanea* gut based on transcriptome sequencing, which produced massive raw data and clean reads. In total, 8,539 DELs and 13,263 DEMs were found, some of which were involved in immune responses. Furthermore, different numbers of Toll/Imd signaling pathway genes were identified and characterized using annotation information and online tools. In addition, we predicted and constructed a lncRNA-mediated ceRNA network of the Toll/Imd signaling pathway. Finally, correlation analysis was performed between DELs or DEMs of the Toll/Imd signaling pathway and most changes in the gut bacterial microbiota. Our data provides new insights into innate immune ceRNAs in *A. viridicyanea* and valuable information for future studies on the interactions between the gut bacterial microbiota and innate immune responses in insects.

## Data Availability

The datasets presented in this study can be found in online repositories. The names of the repository/repositories and accession number(s) can be found in the article/Supplementary Material. Further inquiries can be directed to the first or corresponding authors.
