# Supplementary figures and images for: The lncRNA-mediated ceRNA network of Altica viridicyanea is involved in the regulation of the Toll/Imd signaling pathway under antibiotic treatment
Source: Front Physiol. 2023 Aug 17;14:1244190. doi: 10.3389/fphys.2023.1244190 (PMC10470016; doi:10.3389/fphys.2023.1244190)

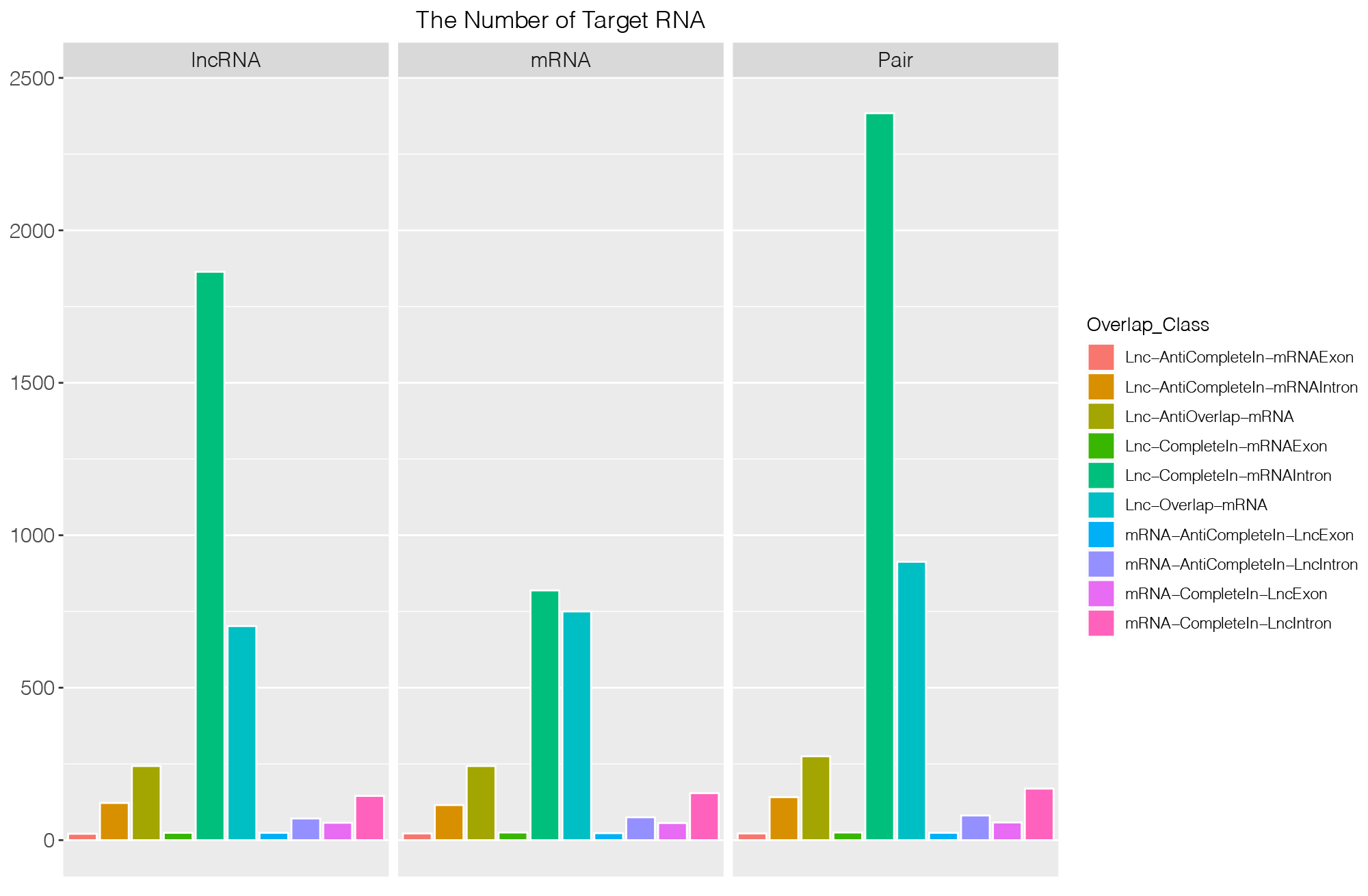

Supplement: Supplementary file 3 [file Image3.TIF]

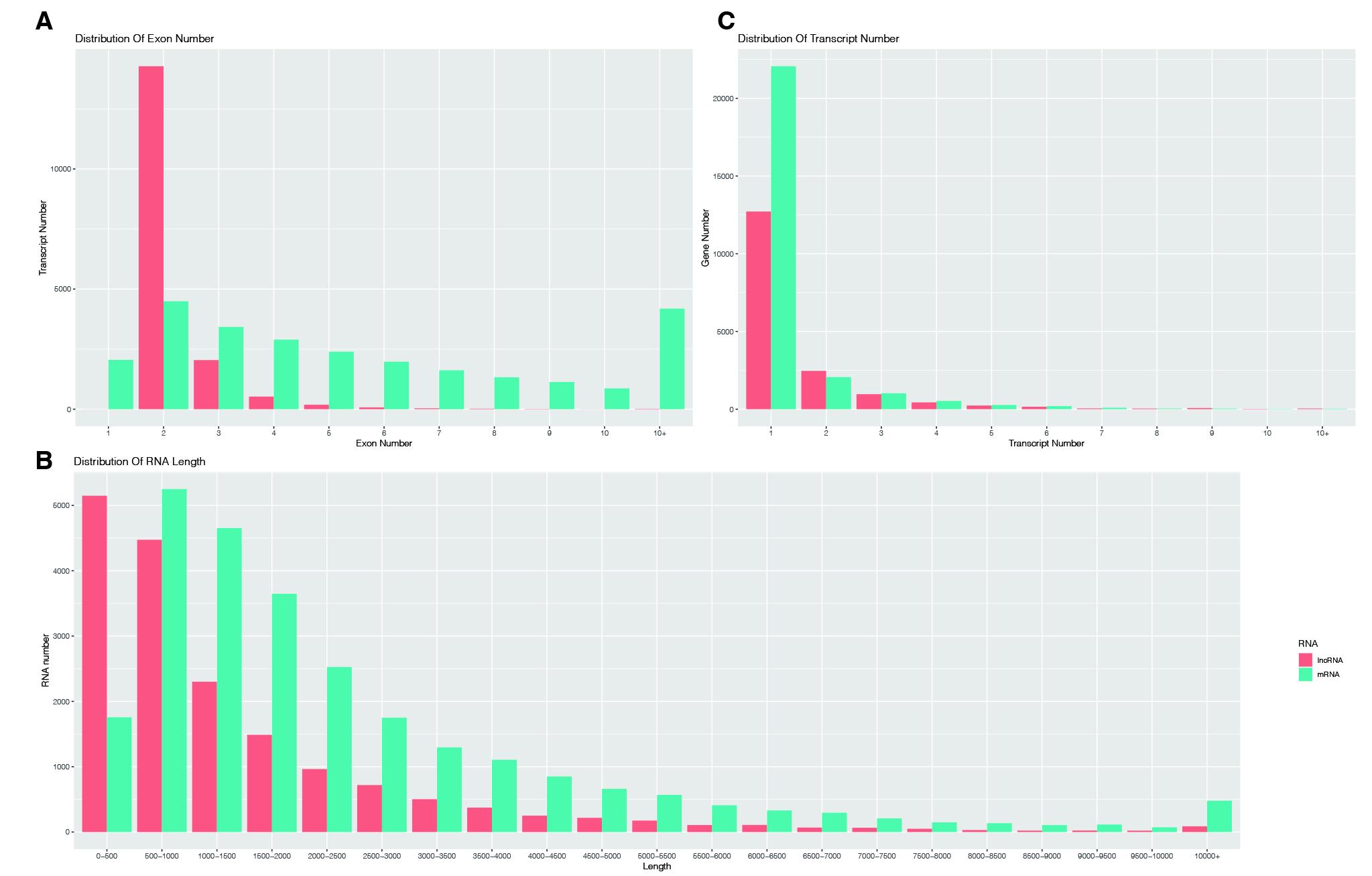

Supplement: Supplementary file 4 [file Image2.TIF]

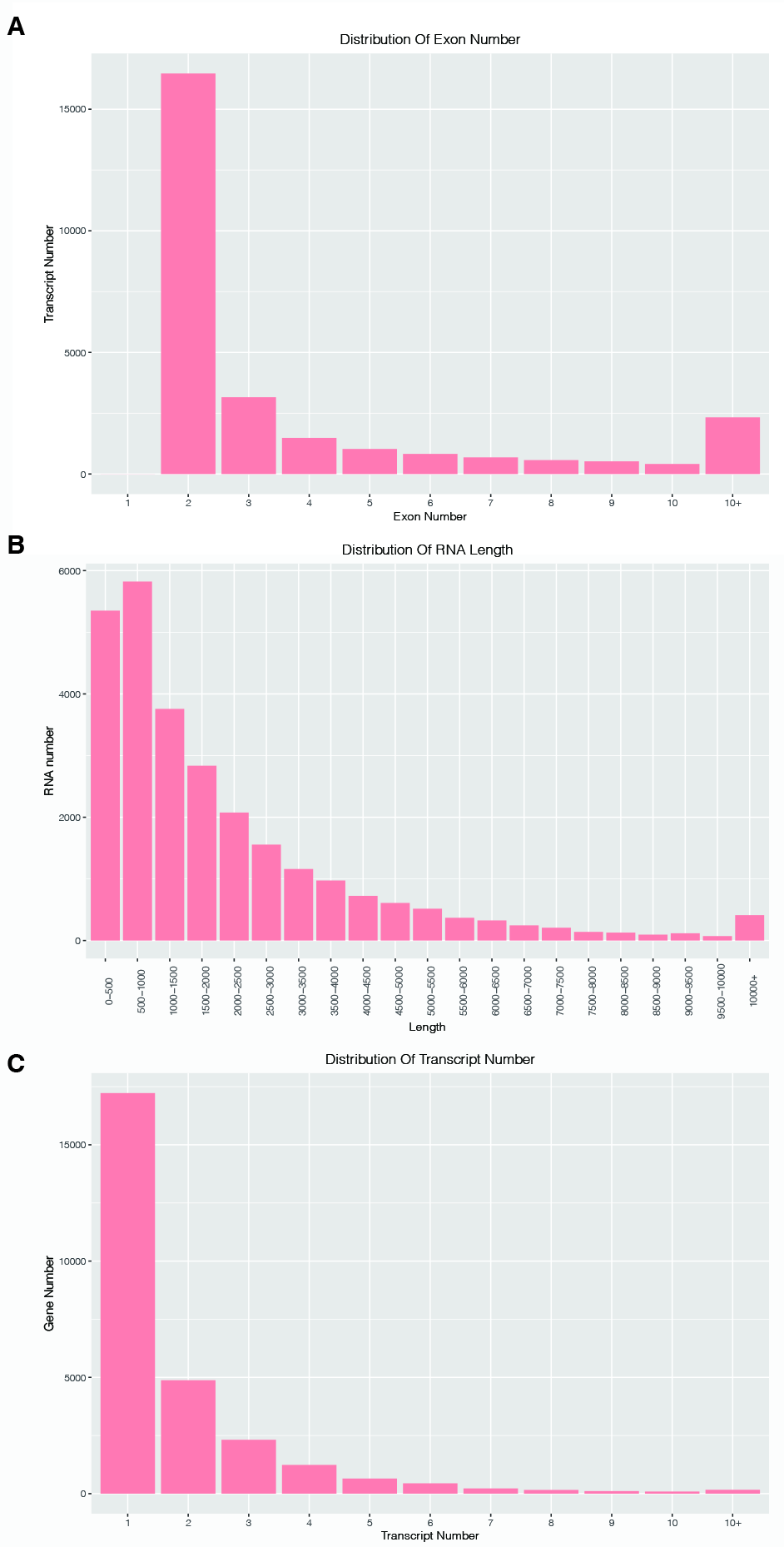

Supplement: Supplementary file 5 [file Image1.TIF]
